# Relative vaccine effectiveness against COVID-19 hospitalisation in persons aged ≥ 65 years: results from a VEBIS network, Europe, October 2021 to July 2023

**DOI:** 10.2807/1560-7917.ES.2024.29.1.2300670

**Published:** 2024-01-04

**Authors:** Mario Fontán-Vela, Esther Kissling, Nathalie Nicolay, Toon Braeye, Izaak Van Evercooren, Christian Holm Hansen, Hanne-Dorthe Emborg, Massimo Fabiani, Alberto Mateo-Urdiales, Ala'a AlKerwi, Susanne Schmitz, Jesús Castilla, Iván Martínez-Baz, Brechje de Gier, Susan Hahné, Hinta Meijerink, Jostein Starrfelt, Baltazar Nunes, Constantino Caetano, Tarik Derrough, Anthony Nardone, Susana Monge, VEBIS-Lot4 working group, VEBIS-Lot 4 working group, James Humphreys, Alexis Sentís, Joris Van Loenhout, Pierre Hubin, Katrine Finderup Nielsen, Chiara Sacco, Daniele Petrone, Patrizio Pezzotti, Itziar Casado, Aitziber Echeverria, Camino Trobajo-Sanmartín, Stijn Andeweg, Anja Bråthen Kristoffersen, Irina Kislaya, Patricia Soares, Carlos Dias, Ausenda Machado

**Affiliations:** 1 National Centre of Epidemiology, Carlos III National Health Institute (ISCIII), Madrid, Spain; 2 Public Health and Epidemiology research group, School of Medicine and Health Sciences, Universidad de Alcalá, Alcalá de Henares, Madrid, Spain; 3 Epiconcept, Paris, France; 4 Vaccine Preventable Diseases and Immunisation, European Centre for Disease Prevention and Control (ECDC), Stockholm, Sweden; 5 Sciensano, Elsene, Belgium; 6 Department of Infectious Disease Epidemiology and Prevention, Statens Serum Institut (SSI), Copenhagen, Denmark; 7 Infectious Diseases Department, Istituto Superiore di Sanità, Rome, Italy; 8 Ministry of Health, Directorate of Health, Service epidemiology and statistics, Luxembourg; 9 Instituto de Salud Pública de Navarra – IdiSNA, Pamplona, Spain; 10 CIBER Epidemiología y Salud Pública (CIBERESP), Spain; 11 Center for Infectious Disease Control, National Institute for Public Health and the Environment (RIVM), Bilthoven, the Netherlands; 12 Norwegian Institute of Public Health (NIPH), Oslo, Norway; 13 Departamento de Epidemiologia, Instituto Nacional de Saúde Doutor Ricardo Jorge, Departamento de Epidemiologia, Lisboa, Portugal; 14 CIBER on Infectious Diseases (CIBERINFEC), Madrid, Spain; 15 The members of the VEBIS-Lot4 working group are listed under Collaborators

**Keywords:** COVID-19, SARS-CoV-2, vaccine effectiveness, hospitalisation, cohort design, electronic health records, multi-country study

## Abstract

To monitor relative vaccine effectiveness (rVE) against COVID-19-related hospitalisation of the first, second and third COVID-19 booster (vs complete primary vaccination), we performed monthly Cox regression models using retrospective cohorts constructed from electronic health registries in eight European countries, October 2021–July 2023. Within 12 weeks of administration, each booster showed high rVE (≥ 70% for second and third boosters). However, as of July 2023, most of the relative benefit has waned, particularly in persons ≥ 80-years-old, while some protection remained in 65–79-year-olds.

Since 2021, the Vaccine Effectiveness, Burden and Impact Studies of coronavirus disease 2019 (COVID-19) and influenza (VEBIS) project monitors vaccine effectiveness (VE) in real-world conditions to inform vaccination programmes in the European Union/European Economic Area (EU/EEA) countries [1]. One project aims to monitor real-time COVID-19 VE using electronic health registries (EHR) in multiple countries, with initial findings previously published [2-4]. We report pooled VE results against hospitalisation due to COVID-19 by number of doses received and time since vaccination in a community-dwelling resident population aged ≥ 65 years between October 2021 and July 2023.

## Design of a monitoring framework to evaluate vaccine effectiveness of COVID-19

The study period covers 22 months, including the sequential circulation of different Omicron BA subvariants and the administration of adapted vaccines against Omicron BA subvariants (Phylogenetic Assignment of Named Global Outbreak (Pango) lineage designation B.1.1.529), in the autumn 2022. See Supplementary Figure S1 for the variation in the proportion of selected severe acute respiratory syndrome coronavirus 2 (SARS-CoV-2) variants over the study period in the participating sites.

Eight sites participated in the study: Denmark, Navarra (Spain), Norway and Portugal from October 2021, Belgium from July 2022, Luxembourg between July 2022 and February 2023, the Netherlands from November 2022 and Italy from December 2022. Retrospective population-cohorts were constructed at each site following a common protocol [5,6]. Individual deterministic linkage was used to cross-match administrative databases with registries for COVID-19 vaccination, SARS-CoV-2 testing, hospitalisations and, in some instances, surveillance data. Estimates of VE were produced monthly to have a near real-time monitoring system of COVID-19 VE and to be able to detect changes in VE for informing public health decisions. To accumulate sufficient events to support VE estimation, each monthly estimate included an observation period of 8 weeks, with a lag of 1 month for data consolidation (i.e. estimates produced in January 2023 covered October–November 2022). This resulted in an overlap in the observation periods, which were rolled 1 month forward for each successive monthly estimate.

The detailed methodology is published elsewhere [5,6]. Briefly, hospitalisation due to COVID-19 was defined as a hospital admission due to a severe acute respiratory infection with a SARS-CoV-2 positive test from 14 days before to 1 day after admission [7] or as COVID-19 as the main diagnosis in admission or discharge records. Vaccination status was time-varying and categorised as: (i) complete primary vaccination administered ≥ 24 weeks earlier (reference category in all models), (ii) first booster administered ≥ 90 days after primary vaccination, (iii) second booster administered ≥ 90 days after the first booster and (iv) third booster administered ≥ 90 days after the second booster. Time after each booster was categorised as: (i) 0 to < 12 weeks, (ii) 12 to < 24 weeks and (iii) ≥ 24 weeks. Time 0 was defined as the end of the induction period, i.e. 14 days after administration of the first dose.

**Figure 1:**
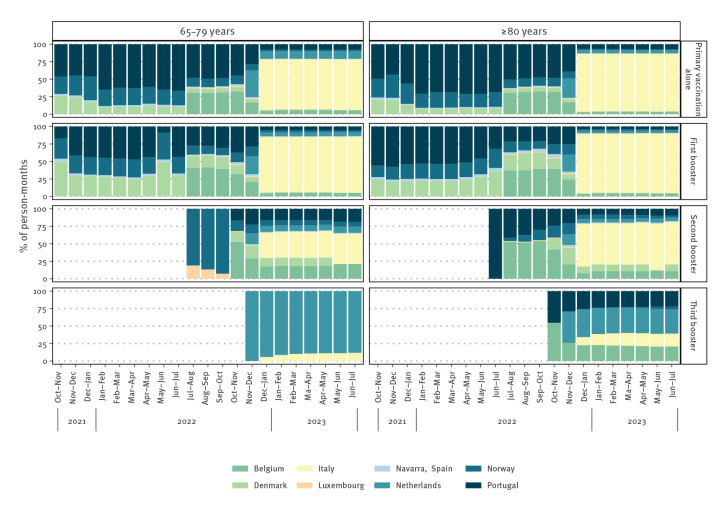
Estimated person-months in VEBIS studies against COVID-19 hospitalisation in persons aged 65–79 and ≥ 80 years, by observation period, vaccination status, age-group and study site, eight European Union/European Economic Area countries^a^, October 2021–July 2023

COVID-19: coronavirus disease 2019; VEBIS: Vaccine Effectiveness (VE), Burden and Impact Studies of COVID-19 and influenza.

^a^ Participation in the study: Belgium (July 2022–July 2023), Denmark (October 2021–July 2023), Italy (December 2022–July 2023), Luxembourg (July 2022–March 2023), the Netherlands (November 2022–July 2023), Norway (October 2021–July 2023), Portugal (October 2021–July 2023) and Spain (Navarra) (October 2021–July 2023).

For each 8-week observation period, follow-up started on the first day and ended at the earliest date of either hospitalisation due to COVID-19, death, emigration outside the country or end of the observation period. Figure 1 shows the cumulative number of person-months by age, vaccination status and study site, reflecting the participation of each site and the timing of booster administration.

We used Cox proportional hazards models to estimate hazard ratios (HR) and 95% confidence intervals (CI) adjusted by sex, age group (in 5-year age bands), previous SARS-CoV-2 infection, comorbidities and other variables as relevant at each site [3,4] We computed relative VE (rVE) as (1-HR) × 100%. Estimates were pooled using Paule-Mandel random-effect meta-analysis [8].

## Relative vaccine effectiveness by time since the last dose

**Figure 2:**
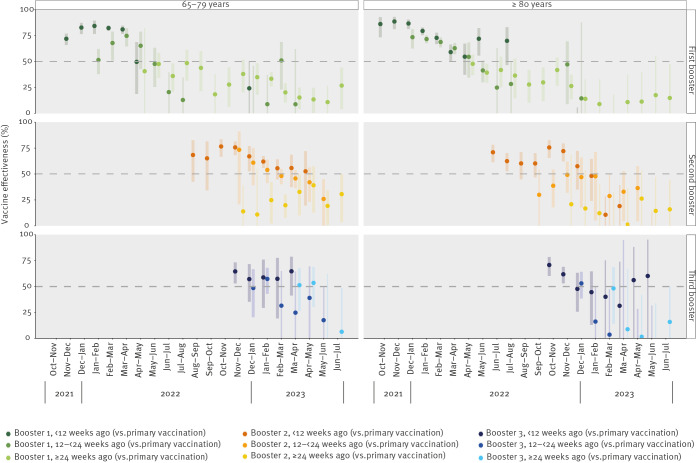
Estimated relative vaccine effectiveness against COVID-19 hospitalisation in persons aged 65–79 and ≥ 80 years in overlapping observation intervals of 8 weeks by number of vaccine doses and time since the latest dose, eight European Union/European Economic Area countries^a^, October 2021–July 2023

^a^ Participation in the study: Belgium (July 2022–July 2023), Denmark (October 2021–July 2023), Italy (December 2022–July 2023), Luxembourg (July 2022–March 2023), the Netherlands (November 2022–July 2023), Norway (October 2021–July 2023), Portugal (October 2021–July 2023) and Spain (Navarra) (October 2021–July 2023).

In persons aged ≥ 80 years, rVE of the first booster administered < 12 weeks earlier (Figure 2, Table 1), declined from 86% (95% CI: 73–93) in October–November 2021 to 55% (95% CI: 37–67) in April–May 2022. Estimates were inconsistent thereafter, possibly reflecting some individuals receiving the first booster during spring vaccination campaigns in 2022 (Belgium and Portugal). The relative VE of the first booster administered 12 to < 24 weeks earlier decreased from 73% (95% CI: 63–81) in December 2021–January 2022 to ≤ 50% in May–June 2022, with high uncertainty afterwards. When the first booster was administered ≥ 24 weeks earlier, the rVE was ≤ 50% throughout the study period and dropped to 15% (95% CI: −38 to 48) in June–July 2023. Similar estimates and overall trends were observed in persons in the age group of 65–79-years-old (Table 2).

**Table 1:** Estimated relative vaccine effectiveness against COVID-19 hospitalisation in persons aged ≥ 80 years in overlapping observation intervals of 8 weeks by number of vaccine doses and time since the latest dose, eight European Union/European Economic Area countries^a^, October 2021–July 2023

Period	Time since the last dose								
	< 12 weeks			12 to < 24 weeks			≥ 24 weeks		
	rVE (%)	95% CI	Countries	rVE (%)	95% CI	Countries	rVE (%)	95% CI	Countries
First booster^b^									
1 Oct–25 Nov 2021	86	73 to 93	DK, ES, NO, PT	NA			NA		
1 Nov–26 Dec 2021	89	81 to 93		NA			NA		
1 Dec 2021–25 Jan 2022	87	81 to 90		73	63 to 81	DK, ES, PT	NA		
1 Jan–25 Feb 2022	79	75 to 83		72	68 to 75	DK, ES, NO, PT	NA		
1 Feb–28 Mar 2022	73	67 to 78		69	64 to 73		NA		
1 Mar–25 Apr 2022	59	50 to 66	DK, NO, PT	63	57 to 68		50	2 to 75	DK, ES, NO, PT
1 Apr–26 May 2022	55	37 to 67		55	34 to 68		48	37 to 57	
1 May–25 Jun 2022	72	56 to 82	PT	41	30 to 51		39	29 to 48	
1 Jun–26 Jul 2022	NA			25	−53 to 63	DK, NO, PT	42	25 to 55	
1 Jul–25 Aug 2022	70	46 to 83	PT	28	−3 to 50	BE, DK, NO, PT	36	15 to 53	
1 Aug–25 Sep 2022	NA			NA			28	10 to 42	BE, DK, ES, NO, PT
1 Sep–26 Oct 2022	NA			NA			30	12 to 44	BE, ES, LU, NO, PT
1 Oct–25 Nov 2022	NA			NA			42	27 to 54	BE, DK, ES, LU, NO, PT
1 Nov–26 Dec 2022	NA			47	9 to 69	NO, PT	26	13 to 37	BE, DK, ES, LU, NL, NO, PT
1 Dec 2022–25 Jan 2023	−39	−104 to 6	IT	14	−502 to 88	IT, NO	14	−10 to 33	BE, DK, ES, IT, LU, NL, NO, PT
1 Jan–25 Feb 2023	NA			−79	−179 to −15	IT	9	−23 to 33	
1 Feb–28 Mar 2023	NA			NA			-8	−42 to 18	BE, DK, IT, LU, NL, NO, PT
1 Mar–25 Apr 2023	NA			−82	−206 to −8	IT	11	−27 to 37	BE, DK, IT, NL, NO, PT
1 Apr–26 May 2023	NA			−166	−342 to −60	IT	11	−31 to 40	
1 May–25 Jun 2023	NA			NA			18	−53 to 56	BE, IT, NL, NO, PT
1 Jun–26 Jul 2023	NA			NA			15	−38 to 48	BE, ES, IT, NL, NO, PT
Second booster^c^									
1 Jun–26 Jul 2022	71	61 to 78	DK, PT	NA			NA		
1 Jul–25 Aug 2022	62	53 to 70	DK, NO, PT	NA			NA		
1 Aug–25 Sep 2022	60	45 to 71	BE, DK, NO, PT	NA			NA		
1 Sep–26 Oct 2022	60	47 to 70	BE, NO, PT	30	−7 to 54	BE, LU, PT	NA		
1 Oct–25 Nov 2022	76	65 to 83		39	18 to 54	BE, LU, NO, PT	NA		
1 Nov–26 Dec 2022	72	62 to 79	BE, ES, NL, NO, PT	49	32 to 62	BE, LU, NL, NO, PT	21	−100 to 69	LU, NL
1 Dec 2022–25 Jan 2023	58	35 to 72	BE, ES, IT, NL, NO, PT	47	17 to 66	BE, IT, LU, NL, NO, PT	17	−49 to 53	BE, IT, LU, NL, PT
1 Jan–25 Feb 2023	48	24 to 65		48	5 to 71	BE, IT, NL, NO, PT	12	−29 to 40	BE, IT, LU, NL, NO, PT
1 Feb–28 Mar 2023	11	−12 to 29	IT, LU	29	−2 to 50	BE, ES, IT, LU, NL, NO, PT	−2	−55 to 32	
1 Mar–25 Apr 2023	19	−8 to 39	DK, IT	33	5 to 53	BE, DK, ES, IT, NL, NO, PT	1	−53 to 36	BE, IT, NL, NO, PT
1 Apr–26 May 2023	NA			38	1 to 60		26	−25 to 56	BE, DK, ES, IT, NL, NO, PT
1 May–25 Jun 2023	NA			−16	−57 to 14	IT	14	−39 to 47	BE, ES, IT, NL, NO, PT
1 Jun–26 Jul 2023	NA			NA			16	−26 to 44	BE, DK, ES, IT, NL, NO, PT
Third booster^d^									
1 Oct–25 Nov 2022	71	60 to 79	BE	NA			NA		
1 Nov–26 Dec 2022	62	53 to 69	BE, NL, PT	NA			NA		
1 Dec 2022–25 Jan 2023	48	26 to 63	BE, IT, NL, PT	53	38 to 64	BE, NL, PT	NA		
1 Jan–25 Feb 2023	44	13 to 65		16	−39 to 49	BE, IT, NL, PT	NA		
1 Feb–28 Mar 2023	40	−45 to 75		3	−77 to 47		48	14 to 69	BE, NL
1 Mar–25 Apr 2023	31	−81 to 74	BE, IT, NL	−1,043	−253,127 to 95		9	−153 to 67	BE, NL, NO, PT
1 Apr–26 May 2023	56	−64 to 88	BE, IT, NO	−1	−42 to 28		1	−67 to 42	BE, IT, NL, PT
1 May–25 Jun 2023	60	−237 to 95		−69	−319 to 32	BE, IT, PT	8	−77 to 34	
1 Jun–26 Jul 2023	NA			NA			16	−42 to 50	

BE: Belgium; CI: confidence interval; DK: Denmark; ES: Spain (Navarra); IT: Italy; LU: Luxembourg; NA: not available; NL: the Netherlands; NO: Norway; PT: Portugal; rVE: relative vaccine effectiveness.

^a^ Participation in the study: Belgium (July 2022–July 2023), Denmark (October 2021–July 2023), Italy (December 2022–July 2023), Luxembourg (July 2022–March 2023), the Netherlands (November 2022–July 2023), Norway (October 2021–July 2023), Portugal (October 2021–July 2023) and Spain (Navarra) (October 2021–July 2023).

^b^ First booster rollout date: Belgium (September 2021), Denmark (October 2021), Italy (September 2021), Luxembourg (July 2021), the Netherlands (November 2021), Norway (October 2021), Portugal (October 2021) and Spain (Navarra) (October 2021).

^c^ Second booster rollout date: Belgium (July 2022), Denmark (September 2022), Italy (April 2022), Luxembourg (April 2022), the Netherlands (February 2022), Norway (June 2022), Portugal (May 2022) and Spain (Navarra) (October 2022).

^d^ Third booster rollout date: Belgium (September 2022), Italy (October 2022), the Netherlands (September 2022), Norway (March 2023) and Portugal (September 2022).

Countries with five events were included in the analysis.

**Table 2:** Estimated relative vaccine effectiveness against COVID-19 hospitalisation in persons aged 65–79 years, in overlapping observation intervals of 8 weeks by number of vaccine doses and time since the last dose, eight European Union/European Economic Area countries^a^, November 2021–July 2023

Period	Time since the last dose								
	< 12 weeks			12 to < 24 weeks			≥ 24 weeks		
	rVE (%)	95% CI	Countries	rVE (%)	95% CI	Countries	rVE (%)	95% CI	Countries
First booster^b^									
1 Nov–26 Dec 2021	72	66 to 77	DK, ES, NO, PT	NA			NA		
1 Dec 2021–25 Jan 2022	83	77 to 87		NA			NA		
1 Jan–25 Feb 2022	84	77 to 89		51	38 to 62	DK, ES, NO, PT	NA		
1 Feb–28 Mar 2022	82	79 to 85		68	51 to 79		NA		
1 Mar–25 Apr 2022	81	77 to 85	DK, NO, PT	75	65 to 82		NA		
1 Apr–26 May 2022	50	19 to 69		65	43 to 79		41	−98 to 82	NO, PT
1 May–25 Jun 2022	NA			48	25 to 63		48	34 to 58	DK, ES, NO, PT
1 Jun–26 Jul 2022	NA			20	−4 to 40	DK, NO, PT	36	21 to 48	
1 Jul–25 Aug 2022	NA			13	−16 to 35	BE, DK, LU, NO, PT	48	32 to 61	BE, DK, ES, LU, NO, PT
1 Aug–25 Sep 2022	NA			NA			44	21 to 60	
1 Sep–26 Oct 2022	NA			NA			18	−71 to 38	
1 Oct–25 Nov 2022	NA			NA			28	6 to 44	
1 Nov–26 Dec 2022	NA			NA			38	20 to 52	BE, DK, ES, LU, NL, NO, PT
1 Dec 2022–25 Jan 2023	24	−15 to 50	DK, IT	-3	−97 to 46	IT, NO	35	18 to 48	BE, DK, ES, IT, LU, NL, NO, PT
1 Jan–25 Feb 2023	NA			9	−32 to 37	IT, NL, NO	33	26 to 40	BE, DK, ES, IT, NL, NO, PT
1 Feb–28 Mar 2023	NA			51	23 to 69	DK, IT, LU, NL, PT	20	10 to 29	BE, DK, ES, IT, LU, NL, NO, PT
1 Mar–25 Apr 2023	NA			9	−119 to 62	DK, IT, PT	15	4 to 25	BE, DK, ES, IT, NL, NO, PT
1 Apr–26 May 2023	NA			NA			15	−1 to 28	BE, DK, IT, NL, NO, PT
1 May–25 Jun 2023	NA			NA			11	−8 to 27	BE, ES, IT, NL, NO, PT
1 Jun–26 Jul 2023	NA			NA			27	4 to 44	
Second booster^c^									
1 Aug–25 Sep 2022	68	42 to 83	NO	NA			NA		
1 Sep–26 Oct 2022	65	34 to 81	LU, NO	NA			NA		
1 Oct–25 Nov 2022	77	66 to 83	LU, NO, PT	NA			NA		
1 Nov–26 Dec 2022	76	68 to 81	BE, ES, NL, NO, PT	73	21 to 91	LU, NL, NO	14	−22 to 39	NL
1 Dec 2022–25 Jan 2023	67	53 to 77	BE, DK, IT, LU, NL, NO, PT	61	39 to 75	IT, LU, NL, NO, PT	11	−76 to 55	IT, NL
1 Jan–25 Feb 2023	62	56 to 67	BE, DK, IT, NL, NO, PT	54	41 to 64	BE, DK, IT, NL, NO, PT	25	2 to 42	DK, IT, NL, NO
1 Feb–28 Mar 2023	56	45 to 64	BE, IT, LU, NL, NO, PT	48	40 to 55	BE, ES, IT, LU, NL, NO, PT	20	8 to 30	IT, LU, NL, NO
1 Mar–25 Apr 2023	56	37 to 69	IT	46	36 to 54	BE, DK, ES, IT, NL, NO, PT	33	10 to 50	BE, IT, NL, NO, PT
1 Apr–26 May 2023	53	19 to 72	ES, IT	42	19 to 59	BE, DK, IT, NL, NO, PT	38	8 to 58	BE, DK, IT, NL, NO, PT
1 May–25 Jun 2023	NA			26	0 to 45	BE, IT, NO, PT	19	1 to 34	BE, DK, ES, IT, NL, NO, PT
1 Jun–26 Jul 2023	NA			NA			31	3 to 50	BE, ES, IT, NL, NO, PT
Third booster^d^									
1 Nov–26 Dec 2022	65	53 to 73	NL	NA			NA		
1 Dec 2022–25 Jan 2023	57	35 to 72	IT, NL	48	20 to 67	NL	NA		
1 Jan–25 Feb 2023	59	29 to 76		57	43 to 68		NA		
1 Feb–28 Mar 2023	57	19 to 78		31	−34 to 65	IT, NL	NA		
1 Mar–25 Apr 2023	65	41 to 79		25	−60 to 65		51	26 to 68	NL
1 Apr–26 May 2023	NA			39	−22 to 69		53	30 to 69	
1 May–25 Jun 2023	NA			17	−35 to 49	IT	0	−168 to 62	IT, NL
1 Jun–26 Jul 2023	NA			NA			6	−71 to 49	

BE: Belgium; CI: confidence interval; DK: Denmark; ES: Spain (Navarra); IT: Italy; LU: Luxembourg; NA: not available; NL: the Netherlands; NO: Norway; PT: Portugal; rVE: relative vaccine effectiveness.

^a^ Participation in the study: Belgium (July 2022–July 2023), Denmark (October 2021–July 2023), Italy (December 2022–July 2023), Luxembourg (July 2022–March 2023), the Netherlands (November 2022–July 2023), Norway (October 2021–July 2023), Portugal (October 2021–July 2023) and Spain (Navarra) (October 2021–July 2023).

^b^ First booster rollout date: Belgium (September 2021), Denmark (October 2021), Italy (September 2021), Luxembourg (July 2021), the Netherlands (November 2021), Norway (October 2021), Portugal (October 2021) and Spain (Navarra) (October 2021).

^c^ Second booster rollout date: Belgium (July 2022), Denmark (September 2022), Italy (April 2022), Luxembourg (April 2022), the Netherlands (February 2022), Norway (June 2022), Portugal (May 2022) and Spain (Navarra) (October 2022).

^d^ Third booster rollout date: Belgium (September 2022), Italy (October 2022), the Netherlands (September 2022), Norway (March 2023) and Portugal (September 2022).

Countries with five events were included in the analysis.

In persons ≥ 80-years-old, rVE of the second booster administered < 12 weeks earlier (Figure 2, Table 1) fluctuated between 71 and 60% from June to October 2022, following spring campaigns (Belgium and Portugal) and between 76 and 72% from October to December 2022, following autumn campaigns and decreasing thereafter. For a second booster 12 to < 24 weeks earlier, rVE varied without a specific time-trend between a maximum of 49% (95% CI: 32–62) and no relative protection (−16%; 95% CI: −57 to 14). No relative protective effect was estimated for a second booster ≥ 24 weeks earlier. In 65–79-year-olds, a similar overall trend was observed, although rVE < 12 weeks earlier remained ≥ 50% until April–May 2023. Estimated rVE of the second booster was 26% (95% CI: 0–45) when administered 12 to < 24 weeks earlier (estimate for May–June 2023) and 31% (95% CI: 3–50) when administered ≥ 24 weeks earlier (estimate for June–July 2023).

The third booster has been recommended to persons aged ≥ 80 years in the Netherlands since September 2022, in Belgium, Italy and Portugal since October 2022, and in Norway since April 2023. When administered < 12 weeks earlier, rVE of the third booster ranged between 71% (95% CI: 60–79) in October–November 2022 and 44% (95% CI: 13–65) in January–February 2023. However, it waned rapidly, with mostly null rVE beyond 12 weeks, albeit with high uncertainty. In persons 65–79-years-old, the third booster had been rolled out only in Italy and the Netherlands since October 2022. Between November 2022 and April 2023, rVE of the third booster administered < 12 weeks earlier ranged between 57 and 65%, although no relative protective effect of the third booster was observed between 12 and < 24 weeks (since February–March 2023) nor beyond 24 weeks (since May–June 2023).

## Discussion

Our results have implications for COVID-19 vaccination roll-out and the monitoring framework. They suggest that successive booster vaccine doses were important for restoring individual protection against COVID-19 hospitalisation in the context of waning effectiveness. However, as of July 2023, most of the relative benefit has waned, particularly in individuals ≥ 80 years, while low relative protection was still observed in persons aged 65–79 years with a first or second booster.

Adapted vaccines against Omicron BA subvariants administered in the autumn 2022 vaccination campaign showed similar rVE when given as the second (76%) or third booster (71%), suggesting that the time since the latest dose is more important for protection than the total number of doses administered. After 12 weeks, rVE declined, especially for the third booster in persons ≥ 80-years-old. These estimates and waning patterns have been observed in other studies [9-13]. Since March 2023, the circulation of the Omicron XBB.1.5 sublineage, with a higher immunity evasion capacity [14], has been increasing, reaching around or above 50% of the isolates in the EU/EEA in July 2023 [15]. This could also partially explain the decrease in the rVE, as other studies suggested [9,16,17].

A multi-country approach can provide representative estimates for the EU/EEA and assess different vaccination roll-out strategies (more details on the roll-out of booster doses by country are provided in Supplementary Table S1). However, estimates may vary due to countries contributing differently and over time, challenging the interpretation of the time-trends. Despite using national databases, a low number of events during certain observation periods, as seen in Supplementary Tables S2–3, resulted in high uncertainty. Additionally, misclassification of hospitalisations not due to COVID-19 cannot be ruled out, despite the specific case-definition. Finally, with increasing number of doses and decreasing coverage of the last dose, comparability across different vaccination statuses regarding vulnerability and past infection can be compromised, despite adjusting for comorbidities, socioeconomic variables and registered SARS-CoV-2 infections (where available). Differential depletion of susceptible persons can also lead to spurious or overestimated waning results [18].

Although the effect of vaccination is likely to differ with respect to prior SARS-CoV-2 infection, we did not stratify by this variable due to suspected high misclassification. This would result from decreasing SARS-CoV-2 testing and increasing use of self-tests along the study period, particularly after the arrival of Omicron in 2021. Therefore, our estimates could be interpreted as an average population effect of the vaccine in country populations with given pre-existing immunity levels. Moreover, because vaccine recommendations are not expected to be made depending on previous infection status we believe our results are relevant from a public health perspective.

To conclude, our results suggest that time since the last booster dose is more important for the risk of hospitalisation than the number of booster doses. This supports seasonal COVID-19 vaccination in population ≥ 65 years at the start of periods with increased expected disease burden. Monitoring focused on the effectiveness of the seasonal vaccine dose is probably more appropriate for the current context.

## Supplementary Data

1269678 Supplementary Material
