# Supplementary material for: Relative vaccine effectiveness against COVID-19 hospitalisation in persons aged ≥ 65 years: results from a VEBIS network, Europe, October 2021 to July 2023
Source: Euro Surveill. 2024 Jan 4;29(1):2300670. doi: 10.2807/1560-7917.ES.2024.29.1.2300670 (PMC10905661; doi:10.2807/1560-7917.ES.2024.29.1.2300670)
Supplement: Supplementary Material [file 23-00670_MONGE_Supplement.pdf]

Supplementary material for the article Relative vaccine effectiveness against COVID-19 hospitalisation in persons aged ≥ 65 years: results from a VEBIS network, Europe, October 2021 and July 2023

This supplementary material is hosted by Eurosurveillance as supporting information alongside the article "Relative vaccine effectiveness against COVID-19 hospitalisation in persons aged ≥ 65 years: results from a VEBIS network, Europe, October 2021 and July 2023", on behalf of the authors, who remain responsible for the accuracy and appropriateness of the content. The same standards for ethics, copyright, attributions and permissions as for the article apply. Supplements are not edited by Eurosurveillance and the journal is not responsible for the maintenance of any links or email addresses provided therein.

Supplementary Table S1. Date of COVID-19 vaccines roll-out by age group, number of booster doses, and study site

	≥80				65-79			
	Complete primary vaccination	Booster 1	Booster 2	Booster 3	Complete primary vaccination	Booster 1	Booster 2	Booster 3
Belgium	21.03.05	21.09.22	22.07.20	22.09.12	21.03.21	21.09.22	22.09.12	
Denmark	20.12.28	21.10.18	22.09.15		21.03.15	21.10.18	22.10.01	
Italy	21.02.01	21.09.27	22.04.08	22.10.17	21.04.07	21.10.08	22.07.11	22.10.17
Luxembourg	21.01.27	21.07.07	22.04.12		21.01.27	21.11.08	22.07.14	
Navarre	21.03.02	21.10.25	22.10.10		21.04.09	21.10.25	22.10.10	
Netherlands	21.02.04	21.11.18	22.02.26	22.09.19	21.04.19	21.12.12	22.03.26	22.10.10
Norway	21.01.31	21.10.05	22.06.29	23.03.24	21.03.07	21.10.05	22.06.29	
Portugal	21.02.03	21.10.11	22.05.16	22.09.06	21.03.30	21.10.11	22.09.06	

Supplementary Table S2. Number of hospitalisations and person-months by number of doses and time since the last dose in ≥80 years old, in overlapping observation intervals of 8 weeks in eight European Union/European Economic Area countries*, October 2021-July-2023

Observation period	Complete primary vaccination ≥24 weeks (events / person-months)	Booster 1 (events / person-months)**			Booster 2 (events / person-months)***			Booster 3 (events / person-months)****		
		≤12 weeks	12-24 weeks	≥24 weeks	≤12 weeks	12-24 weeks	≥24 weeks	≤12 weeks	12-24 weeks	≥24 weeks
October 1 to November 25, 2021	652/ 1,561,710 #¥◊§	37/ 308,023 #¥◊§	-	-						
November 1 to December 26, 2021	751/ 716,577 #¥◊§	180/ 1,124,098 #¥◊§	-	-						
December 1, 2021 to January 25, 2022	620/ 262,137 #¥◊§	824/ 1,723,447 #¥◊§	57/ 23,375 #¥§	-						
January 1 to February 25, 2022	544/ 148,921 #¥◊§	1136/ 1,398,267 #¥◊§	802/ 503,004 #¥◊§	-						
February 1 to March 28, 2022	403/ 119,361 #¥◊§	657/ 599,117 #¥◊§	1877/ 1,271,389 #¥◊§	-						
March 1 to April 25, 2022	220/ 95,555 #¥◊§	207/ 154,349 #◊§	1630/ 1,553,327 #¥◊§	73/ 51,465 #¥◊§						
April 1 to May 26, 2022	175/ 96,037 #¥◊§	44/ 46,477 #◊§	1039/ 1,253,792 #¥◊§	514/ 382,745 #¥◊§	-	-	-	-	-	-
May 1 to June 25, 2022	222/ 102,836 #¥◊§	20/ 21,941 §	368/ 496,691 #¥◊§	1175/ 1,112,868 #¥◊§	-	-	-	-	-	-
June 1 to July 26, 2022	177/ 101,232 #¥◊§	-	88/ 94,915 #◊§	1477/ 1,145,699 #¥◊§	123/ 425,531 #§	-	-	-	-	-
July 1 to August 25, 2022	96/ 109,291 ‡#©¥◊§	15/ 42,839 §	44/ 48,266 ‡#◊§	1987/ 1,915,079 #¥◊§	482/ 1,319,768 #◊§	-	-	-	-	-
August 1 to September 25, 2022	59/ 108,984 ‡#©¥◊§	-	-	1149/ 1,774,973 ‡#¥◊§	327/ 1,179,821 ‡#◊§	-	-	-	-	-
September 1 to October 26, 2022	55/ 98,519 ‡#©¥◊§	-	-	811/ 1,297,159 ‡©¥◊§	201/ 794,411 ‡◊§	80/ 231,325 ‡©§	-	-	-	-

October 1, 2022 to November 25, 2022	51/ 90,233 ‡#©¥◊§	-	-	503/ 801,165 ‡#©¥◊§	136/ 772,923 ‡◊§	195/ 462,708 ‡©◊§	-	167/ 862,107 ‡	-	-
November 1, 2022 ti December 26, 2022	71/ 106,489 ‡#©¥&◊§	-	15/ 10,563 ◊§	716/ 454,074 ‡#©¥&◊§	311/ 645,324 ‡¥&◊§	384/ 320,165 ‡©&◊§	160/ 13,403 ©&	644/ 890,925 ‡&§	-	-
December 1, 2022 to January 25, 2023	204/ 774,124 ‡#$©¥&◊§	27/ 28,838 $	77/ 47,529 $◊	5308/ 3,816,617 ‡#$©¥&◊§	925/ 1,140,714 ‡$¥&◊§	1597/ 1,210,923 ‡$©&◊§	2072/ 1,307,792 ‡$©&§	813/ 830,836 ‡$&§	175/ 202,698 ‡&§	-
January 1 to February 25, 2023	109/ 768,764 ‡#$©¥&◊§	-	21/ 30,389 $	2798/ 3,785,857 ‡#$©¥&◊§	385/ 767,408 ‡$¥&◊§	730/ 1,357,076 ‡$&◊§	1431/ 1,757,523 ‡$©&◊§	317/ 504,094 ‡$&§	697/ 822,392 ‡$&§	-
February 1 to March 28, 2023	64/ 764,927 ‡#$©¥&◊§	-	-	2126/ 3,758,368 ‡#$©&◊§	115/ 340,087 $©	724/ 1,406,235 ‡$©¥&◊§	1486/ 2,126,074 ‡$©&◊§	149/ 309,819 ‡$&§	1246/ 987,494 ‡$&§	22/ 881 ‡&
March 1 to April 25, 2023	52/ 764,364 ‡#$¥&◊§	-	15/ 28,766 $	2173/ 3,772,920 ‡#$&◊§	56/ 171,308 #$	880/ 1,471,988 ‡#$¥&◊§	1785/ 2,562,022 ‡$&◊§	79/ 156,023 ‡$&	931/ 780,728 ‡$&§	290/ 432,680 ‡&◊§
April 1 to May 26, 2023	32/ 762,591 ‡#$¥&◊§	-	16/ 22,045 $	1735/ 3,794,387 ‡#$&◊§	-	371/ 783,065 ‡#$¥&◊§	1844/ 3,449,472 ‡#$¥&◊§	31/ 101,406 ‡$◊	252/ 448,270 ‡$&§	405/ 866,889 ‡$&§
May 1 to June 25, 2023	32/ 757,364 ‡#$¥&◊§	-	-	841/ 3,607,036 ‡$&◊§	-	69/ 308,667 $	860/ 2,981,306 ‡$¥&◊§	19/ 102,924 ‡$◊	55/ 261,970 ‡$§	241/ 627,069 ‡$&§
June 1 to July 26, 2023	23/ 756,047 ‡#$¥&◊§	-	-	419/ 3,629,270 ‡$¥&◊§	-	-	479/ 3,628,433 ‡$#¥&◊§	-	-	128/ 504,708 ‡$&§

rVE = Relative Vaccine Effectiveness; CI = Confidence Interval

*Site participation in the study according to the observation periods: Belgium (July 2022-July 2023), Denmark (October 2021-July 2023), Italy (December 2022-July 2023), Luxembourg (July 2022-March 2023), Navarre (Spain) (October 2021-July 2023), the Netherlands (November 2022-July 2023), Norway (October 2021-July 2023), and Portugal (October 2021-July 2023).

**First booster rollout date by study site where it has been recommended: Belgium (September 2021), Denmark (October 2021), Italy (September 2021), Luxembourg (July 2021), Navarre (Spain) (October 2021), the Netherlands (November 2021), Norway (October 2021), and Portugal (October 2021).

***Second booster rollout date by study site where it has been recommended: Belgium (July 2022), Denmark (September 2022), Italy (April 2022), Luxembourg (April 2022), Navarre (Spain) (October 2022), the Netherlands (February 2022), Norway (June 2022), and Portugal (May 2022).

****Third booster rollout date by study site where it has been recommended: Belgium (September 2022), Italy (October 2022), the Netherlands (September 2022), Norway (March 2023), and Portugal (September 2022).

‡ Belgium reached 5 events and contributed to the estimate
# Denmark reached 5 events and contributed to the estimate
$ Italy reached 5 events and contributed to the estimate
© Luxembourg reached 5 events and contributed to the estimate
¥ Navarre (Spain) reached 5 events and contributed to the estimate
& The Netherlands reached 5 events and contributed to the estimate
◊ Norway reached 5 events and contributed to the estimate
§ Portugal reached 5 events and contributed to the estimate

Supplementary Table S3. Number of hospitalisations and person-months by number of doses and time since the last dose in 65-79 years old, in overlapping observation intervals of 8 weeks in eight European Union/European Economic Area countries*, October 2021-July-2023.

Observation period	Complete primary vaccination ≥24 weeks (events / person-months)	Booster 1 (events / person-months)**			Booster 2 (events / person-months)***			Booster 3 (events / person-months)****		
		≤12 weeks	12-24 weeks	>24 weeks	≤12 weeks	12-24 weeks	>24 weeks	≤12 weeks	12-24 weeks	>24 weeks
October 1 to November 25, 2021	769/ 5,430,687 #¥◊§	-	-	-						
November 1 to December 26, 2021	1,141/ 3,627,044	161/ 1,196,736 #¥◊§	-	-						
December 1, 2021 to January 25, 2022	766/ 1,261,165 #¥◊§	518/ 3,594,550 #¥◊§	-	-						
January 1 to February 25, 2022	515/ 386,220 #¥◊§	997/ 4,724,908 #¥◊§	432/ 256,825 #¥◊§	-						
February 1 to March 28, 2022	340/ 266,654 #¥◊§	941/ 3,226,817 #¥◊§	1028/ 1,674,165 #¥◊§	-						
March 1 to April 25, 2022	176/ 206,324 #¥◊§	248/ 1,124,660 #◊§	914/ 3,440,274 #¥◊§	-						
April 1 to May 26, 2022	70/ 224,508 #¥◊§	47/ 197,431 #◊§	773/ 4,527,698 #¥◊§	46/ 132,006 ◊§	-	-	-	-	-	-
May 1 to June 25, 2022	76/ 263,982 #¥◊§	-	452/ 2,988,196 #¥◊§	459/ 2,003,695 #¥◊§	-	-	-	-	-	-
June 1 to July 26, 2022	59/ 303,450 #¥◊§	-	135/ 756,136 #◊§	1140/ 4,130,625 #¥◊§	-	-	-	-	-	-
July 1 to August 25, 2022	41/ 356,594 ‡#©¥◊§	-	86/ 269,417 ‡#©◊§	1846/ 8,670,660 ‡#©¥◊§	-	-	-	-	-	-
August 1 to September 25, 2022	25/ 370,298 ‡#©¥◊§	-	-	1132/ 8,678,096 ‡#©¥◊§	27/ 177,640 ◊	-	-	-	-	-
September 1 to October 26, 2022	23/ 345,006 ‡#©¥◊§	-	-	908/ 6,655,844 ‡#©¥◊§	47/ 502,355 ©◊	-	-	-	-	-

October 1, 2022 to November 25, 2022	25/<br>298,826<br>‡#©¥◊§	-	-	517/<br>3,640,592<br>‡#©¥◊§	131/<br>3,775,740<br>©◊§	-	-	-	-	-
November 1, 2022 ti December 26, 2022	45/<br>344,948<br>‡#©¥&◊§	-	-	732/<br>1,914,955<br>‡#©¥&◊§	415/<br>3,970,769<br>‡¥&◊§	249/<br>281,285<br>©&◊	108/<br>9,377<br>&	374/<br>66,422<br>&	-	-
December 1, 2022 to January 25, 2023	90/<br>1,694,435<br>‡#$©¥&◊§	24/<br>90,719<br>#$	65/<br>127,341<br>$◊	4101/<br>10,990,371<br>‡#$©¥&◊§	797/<br>4,991,484<br>‡#$©&◊§	1106/<br>2,454,579<br>$©&◊§	481/<br>387,445<br>$&	454/<br>194,097<br>$&	90/<br>12,734<br>&	-
January 1 to February 25, 2023	56/<br>1,699,564<br>‡#$©¥&◊§	-	37/<br>102,722<br>$&◊	2239/<br>10,813,752<br>‡#$¥&◊§	322/<br>2,913,238<br>‡#$&◊§	682/<br>5,068,885<br>‡#$&◊§	512/<br>1,121,563<br>#$&◊	200/<br>226,127<br>$&	326/<br>48,132<br>&	-
February 1 to March 28, 2023	42/<br>1,696,136<br>‡#$©¥&◊§	-	30/<br>184,047<br>#$©&§	1926/<br>10,756,367<br>‡#$©¥&◊§	124/<br>1,243,819<br>‡$©&◊§	741/<br>5,807,923<br>‡$©¥&◊§	677/<br>1,994,633<br>$©&◊	82/<br>194,839<br>$&	693/<br>139,058<br>$&	-
March 1 to April 25, 2023	27/<br>1,703,449<br>‡#$¥&◊§	-	21/<br>184,786<br>#$§	1852/<br>10,800,531<br>‡#$¥&◊§	39/<br>468,487<br>$	827/<br>5,786,667<br>‡#$¥&◊§	912/<br>4,040,830<br>‡$&◊§	34/<br>131,369<br>$&	562/<br>203,633<br>$&	101/<br>18,556<br>&
April 1 to May 26, 2023	24/<br>1,710,882<br>‡#$¥&◊§	-	-	1495/<br>10,856,270<br>‡#$&◊§	15/<br>178,357<br>$¥	346/<br>2,904,351<br>‡#$&◊§	1055/<br>7,462,232<br>‡#$&◊§	-	131/<br>221,847<br>$&	190/<br>54,252<br>&
May 1 to June 25, 2023	16/<br>1,686,000<br>‡#$¥&◊§	-	-	797/<br>10,768,240<br>‡$¥&◊§	-	77/<br>1,079,110<br>‡$◊§	521/<br>7,877,285<br>‡#$¥&◊§	-	20/<br>176,794<br>$	123/<br>153,493<br>$&
June 1 to July 26, 2023	13/<br>1,692,300<br>‡#$¥&◊§	-	-	327/<br>10,254,502<br>‡$¥&◊§	-	-	256/<br>6,533,779<br>‡$¥&◊§	-	-	56/<br>239,879<br>$&

rVE = Relative Vaccine Effectiveness; CI = Confidence Interval

*Site participation in the study according to the observation periods: Belgium (July 2022-July 2023), Denmark (October 2021-July 2023), Italy (December 2022-July 2023), Luxembourg (July 2022-March 2023), Navarre (Spain) (October 2021-July 2023), the Netherlands (November 2022-July 2023), Norway (October 2021-July 2023), and Portugal (October 2021-July 2023).

**First booster rollout date by study site where it has been recommended: Belgium (September 2021), Denmark (October 2021), Italy (October 2021), Luxembourg (November 2021), Navarre (Spain) (October 2021), the Netherlands (December 2021), Norway (October 2021), and Portugal (October 2021).

***Second booster rollout date by study site where it has been recommended: Belgium (September 2022), Denmark (October 2022), Italy (July 2022), Luxembourg (July 2022), Navarre (Spain) (October 2022), the Netherlands (March 2022), Norway (June 2022), and Portugal (September 2022).

****Third booster rollout date by study site where it has been recommended: Italy (not a formal recommendation by the authorities, but available to the age-group) (October 2022), and the Netherlands (October 2022).

‡ Belgium reached 5 events and contributed to the estimate
# Denmark reached 5 events and contributed to the estimate
$ Italy reached 5 events and contributed to the estimate
© Luxembourg reached 5 events and contributed to the estimate
¥ Navarre (Spain) reached 5 events and contributed to the estimate
& The Netherlands reached 5 events and contributed to the estimate
◊ Norway reached 5 events and contributed to the estimate
§ Portugal reached 5 events and contributed to the estimate

Supplementary Figure S1. Proportion of Omicron variant sublineages out of the SARS-CoV-2 samples sequenced by study site and epidemiological week throughout the study period (October’21-July’23)

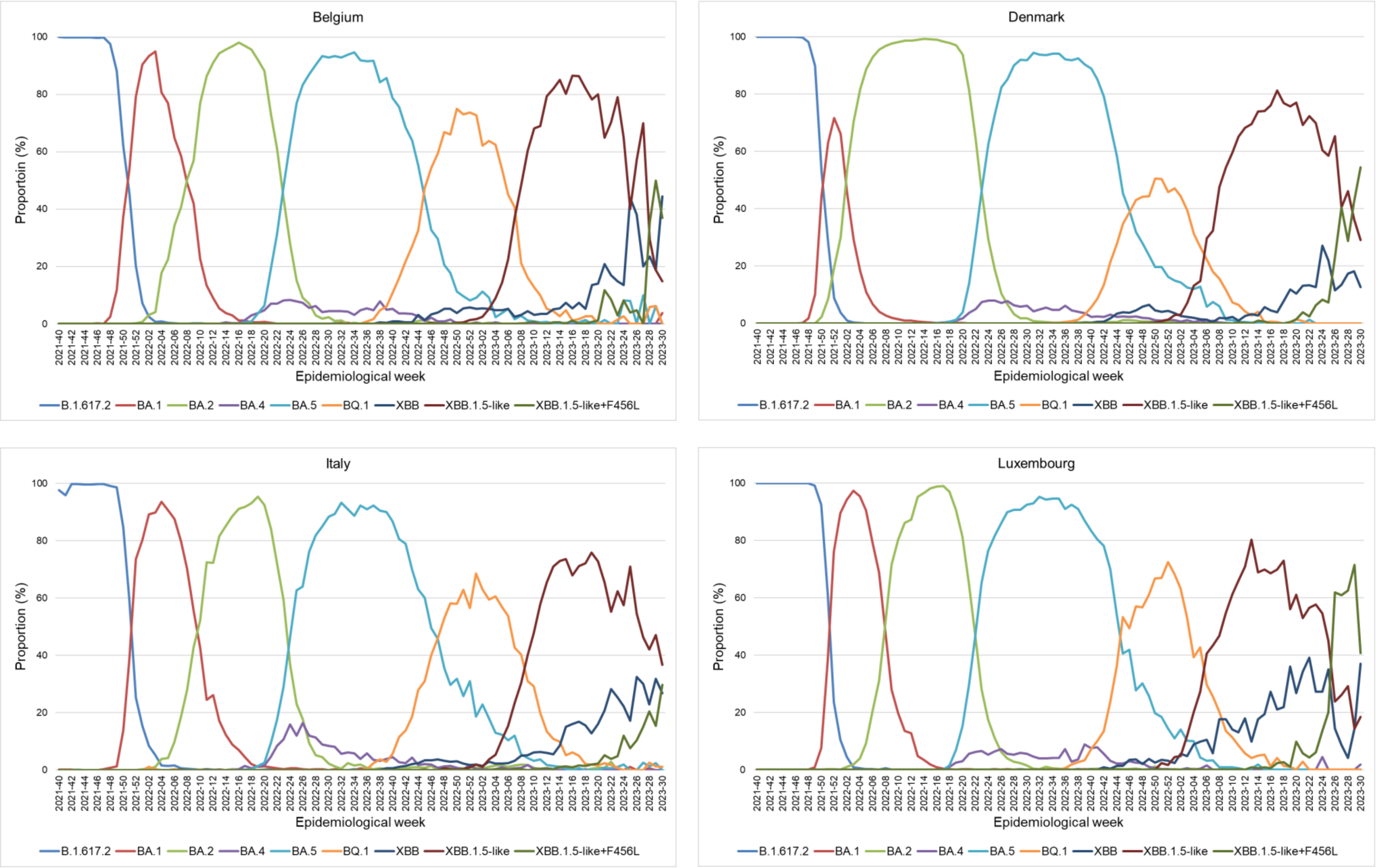

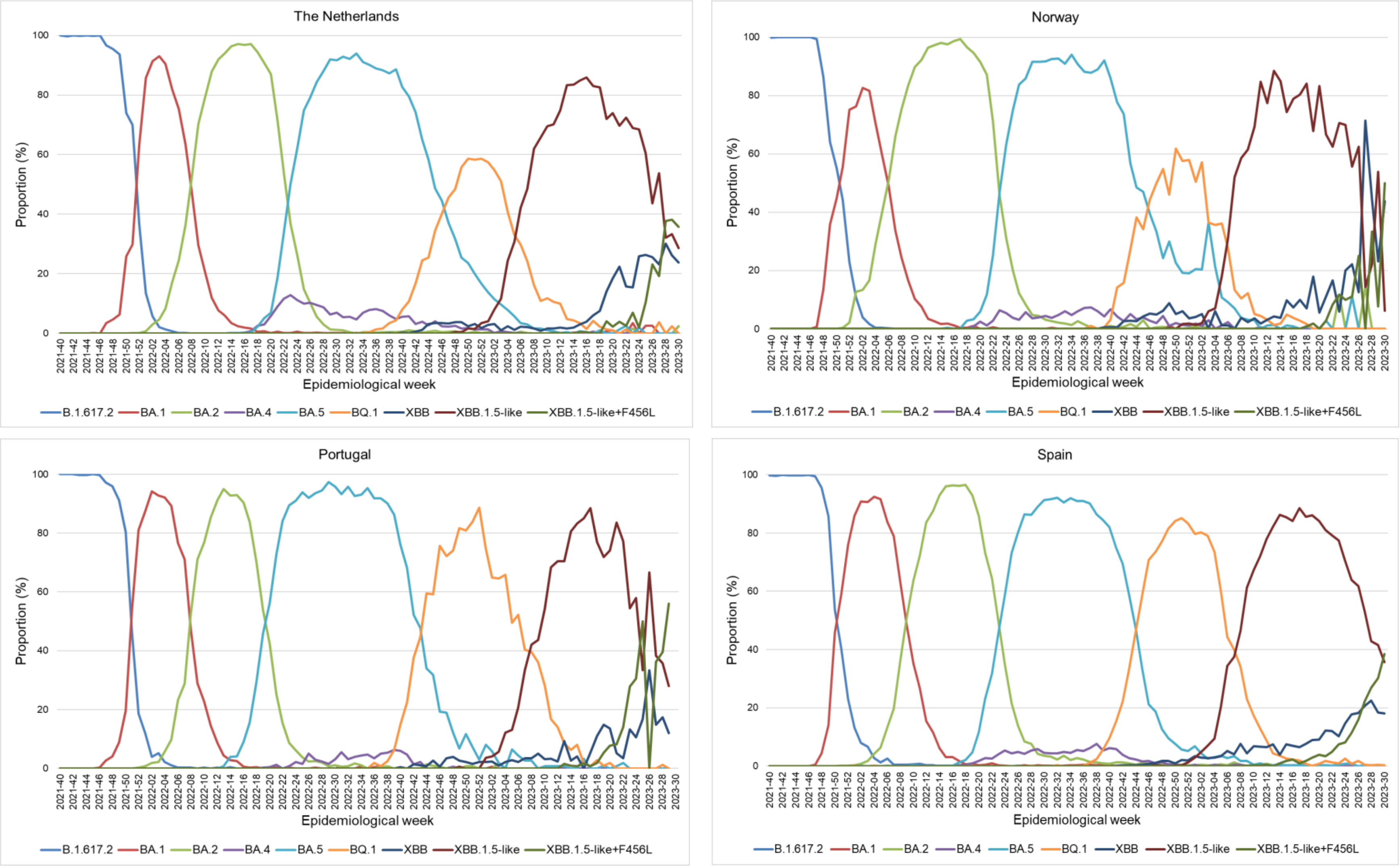

Source: European Centre for Disease Prevention and Control. Data on SARS-CoV-2 variants in the EU/EEA. (2023, November 17) [Internet]. 2023 [cited 2023 Dec 6]. Available from: *https://www.ecdc.europa.eu/en/publications-data/data-virus-variants-covid-19-eueea*
